# Sustainability of large-scale implementation of shared decision making with the SHARE TO CARE program

**DOI:** 10.3389/fneur.2022.1037447

**Published:** 2022-11-23

**Authors:** Constanze Stolz-Klingenberg, Claudia Bünzen, Marie Coors, Charlotte Flüh, Nils G. Margraf, Kai Wehkamp, Marla L. Clayman, Fueloep Scheibler, Felix Wehking, Jens Ulrich Rüffer, Wiebke Schüttig, Leonie Sundmacher, Michael Synowitz, Daniela Berg, Friedemann Geiger

**Affiliations:** ^1^National Competency Center for Shared Decision Making, University Hospital Schleswig-Holstein, Kiel, Germany; ^2^Chair of Health Economics, Technical University of Munich, Munich, Germany; ^3^Department of Neurosurgery, University Hospital Schleswig-Holstein, Kiel, Germany; ^4^Department of Neurology, University Hospital Schleswig-Holstein, Kiel, Germany; ^5^Department of Internal Medicine I, University Hospital Schleswig-Holstein, Kiel, Germany; ^6^Department of Medical Management, MSH Medical School Hamburg, Hamburg, Germany; ^7^Center for Healthcare Organization and Implementation Research (CHOIR), Veterans Administration, Bedford, MA, United States; ^8^Department of Population and Quantitative Health Sciences, University of Massachusetts Chan Medical School, Worcester, MA, United States; ^9^Department of Emergency Medicine, University Hospital Jena, Jena, Germany; ^10^TakePart Media + Science GmbH, Cologne, Germany; ^11^Department of Psychology, MSH Medical School Hamburg, Hamburg, Germany

**Keywords:** shared decision making (SDM), sustainability, SDM implementation, decision aids, training of physicians, patient activation

## Abstract

**Introduction:**

SHARE TO CARE (S2C) is a comprehensive implementation program for shared decision making (SDM). It is run at the University Hospital Schleswig-Holstein (UKSH) in Kiel, Germany, and consists of four combined intervention modules addressing healthcare professionals and patients: (1) multimodal training of physicians (2) patient activation campaign including the ASK3 method, (3) online evidence-based patient decision aids (4) SDM support by nurses. This study examines the sustainability of the hospital wide SDM implementation by means of the Neuromedical Center comprising the Departments of Neurology and Neurosurgery.

**Methods:**

Between 2018 and 2020, the S2C program was applied initially within the Neuromedical Center: We implemented the patient activation campaign, trained 89% of physicians (*N* = 56), developed 12 patient decision aids and educated two decision coaches. Physicians adjusted the patients' pathways to facilitate the use of decision aids. To maintain the initial implementation, the departments took care that new staff members received training and decision aids were updated. The patient activation campaign was continued. To determine the sustainability of the initial intervention, the SDM level after a maintenance phase of 6–18 months was compared to the baseline level before implementation. Therefore, in- and outpatients received a questionnaire *via* mail after discharge. The primary endpoint was the “Patient Decision Making” subscale of the Perceived Involvement in Care Scale (PICS_PDM_). Secondary endpoints were an additional scale measuring SDM (CollaboRATE), and the PrepDM scale, which determines patients' perceived health literacy while preparing for decision making. Mean scale scores were compared using *t*-tests.

**Results:**

Patients reported a significantly increased SDM level (PICS_PDM_ *p* = 0.02; Hedges' *g* = 0.33; CollaboRATE *p* = 0.05; Hedges' *g* = 0.26) and improved preparation for decision making (PrepDM *p* = 0.001; Hedges' *g* = 0.34) 6–18 months after initial implementation of S2C.

**Discussion:**

The S2C program demonstrated its sustainability within the Neuromedical Center at UKSH Kiel in terms of increased SDM and health literacy. Maintaining the SDM implementation required a fraction of the initial intensity. The departments took on the responsibility for maintenance. Meanwhile, an additional health insurance-based reimbursement for S2C secures the continued application of the program.

**Conclusion:**

SHARE TO CARE promises to be suitable for long-lasting implementation of SDM in hospitals.

## Introduction

Patients' satisfaction, successful treatment of diseases and cost-effectiveness are three of the main goals in healthcare, which in some cases are difficult to reconcile. Shared decision making (SDM) as a process of information exchange and negotiation can contribute as an essential lever to achieve these ambitious goals simultaneously (1). To successfully reach a shared decision, a positive and productive collaboration between physician and patient is needed (2). Physicians act as medical experts with their expertise in causes of disease, symptoms, treatment options with possible advantages and disadvantages. Based on the best available evidence they provide the information base for a meaningful physician-patient-conversation (3). The patient acts as an expert on himself, his preferences and his personal circumstances. Patients enrich the information exchange with their thoughts, experiences of disease, risk behavior and expectations—information that is not evident from any medical record (1). The active participation of both leads to a shared decision that is medically justifiable and provides the best fit for the individual patient. Such an exchange on equal terms meets patients need for better information, understanding and involvement in their medical decision making (4–6). In a study with more than 1.500 cancer patients in Germany over 80% of the participants preferred to take a collaborative or active role in decision making underlying the need for increased SDM-practice (7).

In addition to the arguments from the patient's point of view, SDM also offers advantages for physicians as well as the healthcare system: SDM strengthens patients' self-responsibility and knowledge of their disease, so that well-informed patients more rapidly recognize and communicate side-effects. This can be a protective factor against serious treatment complications, especially in complex decision-making situations with a multitude of treatment options, such as in the treatment of multiple sclerosis in neurology (8). Complementary, patients perceive themselves as more self-efficacious (6). In particular through the application of SDM, patients develop more realistic outcome expectations leading to increased patient satisfaction, greater treatment adherence, less decisional regret and less complaints (9–12).

In sum, there are many arguments in favor of SDM. This raises the question: How can SDM be successfully implemented in the hospital initially, and how can it be maintained so that SDM remains a matter of course in daily healthcare?

Only a limited number of SDM-interventions follow a comprehensive approach involving all relevant stakeholders, i.e., patients, physicians, nurses and other healthcare professionals (12). The SHARE TO CARE program (S2C) addresses each of these groups with a dedicated intervention module. Integrating four modules into a comprehensive implementation strategy, the S2C program aims to implement SDM within entire hospitals. The four S2C modules are:

*(1) Training of all physicians*. A minimum of 80% of physicians within each clinical department has to complete a multimodal training composed of an online-training session (13) and two individual feedback sessions based on videotaped patient consultations. Physicians first complete a 1-h online session presenting basic SDM knowledge and several simulations of physician-patient-interactions to demonstrate Do's and Don'ts in SDM. Subsequently, physicians take part in an individual SDM coaching session in a peer group setting (2–5 participants) based on their own videotaped patient consultations. Interaction of increased self-reflection through video excerpts and feedback from colleagues and experienced SDM coaches shall create an encouraging and constructive learning atmosphere. Later, physicians record another consultation and participate in a second small group training to further increase and consolidate their SDM skills. After successful training completion, physicians receive a certificate and education credits by the Physicians Chamber of the Federal State of Schleswig-Holstein.

*(2) Activation of patients*. To increase patients' participation and involvement in medical decision making, every patient receives information how to actively take part in their physician-patient consultations using the ASK-3 approach (14). By distributing various promotion/information material (e.g., SDM video clips on screens, roll ups, posters, flyers, promotional items, SDM web page, paper postcards and screen-based messages and media) inside each department, patients are encouraged to ask specific questions during their consultation to gain deeper understanding of their treatment opportunities.

*(3) Implementation of evidence-based decision aids*. Fostering patients' understanding of their condition and treatment opportunities is also done through online evidence-based Patient Decision Aids (EbPDA). These are developed within each department based on a literature and guideline review, in cooperation with physicians and patients. The evidence research team conducts a systematic review of best available evidence for all treatment opportunities available at the hospital. They also perform needs assessment interviews with patients to align with needs and preferences of patients in the specific decision situations. Methods are based on the German Standard of evidence-based patient information and the methods of evidence generation in patient information (15, 16). Considering EbPDA as a user-oriented interface, text information is complemented with video clips featuring local physicians explaining interventions and local patients who share their experience facing the same decision as the DA user. The process of DA development follows the International Patient Decision Aids Standards (17–19). Each EbPDA undergoes external review. The process of EbPDA development for the S2C program is described in detail elsewhere (20).

As decision aids will never cover every relevant decision within a hospital, clinical experts are to choose topics that are of relevance from a clinical perspective, preference-sensitive as well as sufficiently frequent. These topics are expected to induce a spill-over effect to decisions where no EbPDA is available in terms of a systematic consideration of benefits and harms of each treatment as well as the patient's preferences.

*(4) Integration of nurses as SDM supporters*. At least 80% of the nurses are educated how to integrate SDM within their own work and how to support patients and physicians regarding the application of the abovementioned modules. Beyond this, selected nurses (or physiotherapists, study nurses etc.) are trained as decision coaches to facilitate patients' decision processes with physicians. Training is designed in a similar way as physicians' coaching sessions: During 2 workshop days, healthcare professionals gain further knowledge about SDM, deep insight in the DAs of their specific department or section, and skills to support patients' decision making. Accompanied by the S2C trainer team, nurses complete decision coach training by recording coaching conversations with a patient twice and receiving individual feedback. Decision coaches function as emotional assistance to sensitize patients to unanswered questions and treatment preferences improving the physician-patient-consultation.

By completing all four modules, a department meets the criteria to be awarded with the S2C certificate. The fulfillment of the criteria is reviewed annually.

Initial findings from the hospital-wide implementation of SDM at the UKSH in Kiel indicate that the S2C program is feasible and effective (21).

Beyond the short-term effectiveness of SDM interventions, it is crucial that the effects are long-lasting, or can be maintained with reasonable effort. However, there is very limited research on the sustainability of SDM interventions. In a sample of patients with fibromyalgia, Bieber et al. (2) were able to show a diminished but still significantly enhanced SDM level 1 year after their SDM intervention. In a review by Martinez-Gonzalez et al. (22), only outcome parameters related to SDM—but not the SDM level itself—were reported, e.g., knowledge or perceived information level. No study was found with long-term effects (>3 months). Another study in outpatient asthma practices included data 1 year after implementation, but with poor comparability due to lack of a baseline survey (23).

In summary, there is no conclusive evidence on the sustainability of hospital wide implemented SDM interventions such as the S2C program. Although—given the substantial effort required for large-scale implementation of SDM—such evidence is particularly relevant as only a long-term effective intervention is cost-effective and reasonable for a hospital. To fill this research gap, the aim of this study was to examine the sustainability of the large-scale implementation of SDM with the S2C program at the UKSH in Kiel (24).

## Methods

### Design and setting

To assess the sustainability of the S2C program in terms of a long-lasting increase of the SDM level, we collected data at the Neuromedical Center (Department of Neurology and Department of Neurosurgery) at UKSH in Kiel. Patients were included in 2018 prior to the SDM implementation (baseline *t*_0_), in 2020 immediately after the implementation (*t*_1_) and in 2021 at the end of the funding period of the implementation project, i.e., 6–18 months after implementation (*t*_2_). In this study, data from *t*_2_ and *t*_0_ were compared.

The baseline survey was conducted at a time when neither medical staff nor patients had been informed about the upcoming SDM implementation. At *t*_1_ and *t*_2_, medical staff was informed that the S2C program would be evaluated. However, they were not aware of evaluation measures, the sampling period and, hence, the patients to be included. They had no influence on inclusion of patients. During their consultations, patients were not aware that they might be invited to participate in a study about SDM later.

### Participants

We included adult patients (age 18 and older) who recently had a consultation at the Neuromedical Center at the UKSH in Kiel (inpatients and outpatients). After their discharge, patients were contacted by mail to fill out a questionnaire (see Section 2.4 for details). The study was approved by the Ethics Committee of Faculty of Medicine of Kiel University (reference number A111/18).

### Intervention

#### Initial implementation

Between 2018 and 2020, the S2C program had been applied successfully within the Neuromedical Center: The patient activation campaign had been rolled out, 89% of physicians (N=56) had completed SDM training. 12 patient decision aids had been developed. 2 decision coaches had been educated. Patients' pathways had been adjusted to facilitate the use of decision aids. Implementation took ca. 1.5 years in Neurology and 2 years in Neurosurgery where it was temporarily interrupted by the Covid19 pandemic. Both departments were awarded the S2C certificate indicating full SDM implementation, which is valid 12 months. At *t*_1_ immediately after implementation, the SDM level had significantly increased, as reported elsewhere (21).

#### Maintenance of the implementation

After successful initial implementation, step by step the departments were asked to take on the responsibility for maintenance of the SDM implementation: New physicians should be consecutively trained, and all physicians should participate in further SDM education (twice a year). Other healthcare professionals should be regularly educated. The decision aids should be kept up to date and the patient activation campaign be continued.

### Data collection and outcome measures

Outcome data was collected in a pre–post-design *via* mailed patient questionnaires before (*t*_0_), immediately after (*t*_1_) intervention and at follow-up 6–18 months after (*t*_2_) intervention. Baseline measurements were conducted from July until September 2018 at the Department of Neurology and from August until October 2018 at the Department of Neurosurgery. Long-term post intervention data collection (*t*_2_) took place from May until July 2021 for both departments, shortly before the end of the funding period to cover the longest possible follow up-period. This explains the different time periods between *t*_1_ and *t*_2_ at the Department of Neurology vs. the Department of Neurosurgery. Patients received two mailed reminders if they failed to answer within 4 weeks.

Both inpatients and outpatients were included without exclusion criteria regarding diagnosis or other parameters. Sampling was done as a retrospective and consecutive sample at a certain key date. The overall sample size within the hospital-wide SDM implementation was prescribed by the study protocol (*N* > 1.600 pre and post each) (24). The sample size within each of the 22 departments included within the hospital-wide implementation was determined by its proportion of cases compared to the overall hospital, with a minimum of *N* > 30 per measurement and department. This resulted in a minimum of *N* > 60 in this study in the Neuromedical Center with its two constituting departments.

The primary outcome was the “Patient Decision Making” (PICS_PDM_) subscale of the Perceived Involvement in Care Scale (PICS), a patient reported outcome instrument translated and validated in German (25, 26). It was measured on a scale from 1 = “do not agree at all” to 4 = “totally agree”. PICS_PDM_ can be seen as a key indicator of SDM-based physician-patient interaction and has proven applicable in retrospective studies by mail (27). As secondary outcome, SDM level was assessed using the patient questionnaire collaboRATE (28) (COLL; 3 items; 5 point scale). The Preparation for Decision Making Scale (29) (PrepDM; 10 items; 5 point scale) was used as an indicator of decision-specific health literacy.

In addition, the process of maintaining the SDM implementation was monitored and documented.

### Statistical analyses

For descriptive purposes, data are expressed as mean with standard deviation (SD) and/or 95% confidence interval (CI), unless stated otherwise. A questionnaire was declared evaluable if all questions of the respective subscale were answered. We used *z*-score normalization before pooling the two departments. *t*_0_-baseline and *t*_2_-post-intervention data were compared using independent two-sided *t*-test to examine whether there were significant differences in PICS_PDM_, PrepDM, and COLL. In addition, we performed a multiple regression analysis testing the effect of age, education and gender on PICS_PDM_, PrepDM, and COLL. Effect size was reported using Hedges' *g*. All analyses were performed using STATA 16.1 with a *p*-value < 0.05 considered to indicate statistical significance.

## Results

### Patients' characteristics

During the previously defined sampling period, 109 (63%) of all eligible patients at *t*_0_ mailed back a survey. The sampling period at *t*_2_ happened to contain more eligible patients. 142 of them (59%) sent back their questionnaire. Therefore, both response rates were in the predefined range as described in the study protocol (24).

Details of patients' characteristics are shown in Table 1.

**Table 1:** Sample description.

	***t***_**0**_		***t***_**2**_		**Overall**
	***n***	**%**	***n***	**%**	
**Number of patients**	109		142		251
**Age**
Total responses	107		142		249
18–40 years	6	5.6%	17	12.0%	
41–60 years	40	37.4%	46	32.4%	
61–80 years	53	49.5%	69	48.6%	
Over 80 years	8	7.5%	10	7.0%	
**Gender**
Total responses	99		126		225
Female	45	45.5%	74	58.7%	
Male	54	54.5%	52	41.3%	
**Education**
Total responses	102		140		242
Lower than secondary school certificate	38	37.3%	42	30.0%	
Secondary school certificate	32	31.3%	52	37.2%	
Higher education entrance qualification	28	27.4%	44	31.4%	
Other school qualification	4	4.0%	2	1.4%	

### Transfer to “maintenance mode”

The Neuromedical Center successfully switched into “maintenance mode” regarding the four S2C modules: Until submission of this manuscript, 17 additional physicians completed training and 14 more physicians have entered the training process. Each ward management conducted internal SDM education for nurses at least annually. As a complement to the continued patient activation campaign *via* ASK 3 (flyers, posters, cards), a short video encouraging patients to engage in decision making was made available on the screen at the patient's bedside. The decision aids were regularly reviewed by clinical experts which led to an update of the decision aid for Parkinson's disease.

Beyond the maintenance of the standard S2C modules, the Neuromedical Center proactively expanded its SDM activity: an additional SDM consultation service by specifically trained staff was introduced at the epilepsy outpatient ward. Complementary, the Center engaged in scientific and public relations activity featuring the patient-centeredness as proven by the certified SDM implementation.

### Sustained effects

The newly obtained long-term data from this study revealed a significant increase of the SDM level 6–18 months after the intervention [*z*-score standardized PICS_PDM_: *M*__*t*_0_ = −0.19 (SD = 1.04); *M*__*t*_2_ = 0.14 (SD = 0.95); *p* = 0.02]. The effect size Hedges' *g* = 0.33 indicates a small effect (30).

Patients reported an improved preparation for their treatment decision [PrepDM: *M*__*t*_0_ = −0.20 (SD = 1.00); *M*__*t*_2_ = 0.14 (SD = 0.96); *p* = 0.001; Hedges' *g* = 0.34]. In addition, patients experienced a better collaboration with physicians [Coll: *M*__*t*_0_ = −0.15 (SD = 0.98); *M*__*t*_2_ = 0.11 (SD = 1.00); *p* = 0.05; Hedges' *g* = 0.26] (see Table 2).

**Table 2:** Endpoints before and after implementation (original and *z*-score standardized values).

	**Original values**				***z*** **-score standardized values**				***p***	**Hedges' g**
	*t*_0_		*t*_2_		*t*_0_		*t*_2_			
	***M***	**SD**	***M***	**SD**	***M***	**SD**	***M***	**SD**		
PICS_PDM_	2.65	0.92	2.92	0.85	−0.19	1.04	0.14	0.95	0.02	0.33
COLL	3.15	1.29	3.59	1.26	−0.15	0.98	0.11	1.00	0.05	0.26
PrepDM	3.63	1.12	3.92	1.16	−0.20	1.00	0.13	0.96	0.01	0.34

To examine potential influence of age, gender or education on the primary endpoint PICS_PDM_, we performed a multiple regression analysis. Results indicated that apart from the intervention itself (*p* = 0.05), younger patients reported significantly higher levels of SDM (see Table 3). Table 3 displays results of additional multiple regression analysis on the potential impact of age, gender and educational level on the secondary endpoints COLL and PrepDM.

**Table 3:** Multiple linear regression analysis of the effect of time point of measurement, age, sex, and educational level on the primary endpoint “patient participation in decision making” (PICS_PDM_) and on secondary endpoints CollaboRATE and PrepDM.

	**Dependent variables**		
	**PICS_PDM_**	**COLL**	**PrepDM**

	**Regression coefficient (SD)**	**Regression coefficient (SD)**	**Regression coefficient (SD)**
**Time point of measurement**			
Baseline *t*_0_ (reference group)			
Follow up *t*_2_	0.29^*^ (0.14)	0.26 (0.14)	0.39^**^ (0.15)
**Age (years)**			
18–40 (reference group)			
41–60	−0.62^*^ (0.25)	−0.61^*^ (0.25)	−0.50 (0.26)
Over 60	−0.62^**^ (0.24)	−0.43 (0.24)	−0.38 (0.25)
**Sex**			
Female (reference group)			
Male	−0.11 (0.14)	−0.14 (0.14)	−0.07 (0.15)
**Highest educational level attained**			
Lower than secondary school certificate (reference group)			
Secondary school certificate	0.09 (0.17)	−0.10 (0.16)	−0.10 (0.18)
Higher education entrance qualification	−0.05 (0.17)	−0.15 (0.18)	−0.22 (0.18)
Regression constant	0.44 (0.26)	0.42 (0.27)	0.26 (0.28)
*R* ^2^	0.08	0.06	0.07
*R*^2^ adj.	0.05	0.04	0.04
*n* (*t*_0_)	85	88	83
*n* (*t*_1_)	112	115	110

* *p* < 0.05;

** *p* < 0.01.

## Discussion and conclusion

It had already been shown that the S2C program is feasible and can have positive effects on the SDM level right after full implementation: Immediately after the end of implementation, patients felt significantly more involved and better informed and prepared for decision-making (21). At that time, the healthcare professionals were trained recently and the learning content was fresh in their minds, the decision aids had been newly developed with a great deal of commitment from physicians, and patient activation had just been set up. In short, SDM was on everyone's agenda.

This new study is the first exploring the sustainability of a full S2C implementation in a hospital. The question was, how does the SDM level evolve months after the end of the initial implementation? Do departments deliberately invest resources to maintain the implementation, and does the effect on patient-reported SDM level and health literacy persist?

The results of this study show: Even 6–18 months after the end of the initial implementation of the S2C intervention, i.e., 2.5–3 years after the first physician has been trained, the SDM level is still significantly increased. Patients continue to perceive themselves as significantly more involved than before the intervention as indicated coherently by two different SDM measures, PICS and CollaboRATE. In addition, patients reported a higher health literacy while preparing for a decision. As patients were recruited regardless of their diagnosis, from acute and aftercare, from inpatient and outpatient care, in neurology and neurosurgery, these results seem exceptionally representative.

The sustained intervention effects can be attributed to at least two factors. On the one hand, it may be assumed that the effects from the initial intervention in terms of skills and attitudes have persisted among the healthcare professionals, and that they adhered to the procedures and pathways that were adapted to allow for SDM. In line with Légaré et al. (12), the comprehensiveness and intensity of a multifaceted program like S2C in combination with the fact that nearly all departments at the hospital in Kiel were transformed into SDM clinics simultaneously is supposed to have had enough impact to stimulate a cultural change that is more robust than a rather superficial adoption of SDM-related communication skills. On the other hand, the maintenance activity from the Neuromedical Center is regarded as important to secure sustainability. Within every hospital, let alone a university hospital, the turnover within the healthcare personnel requires constant introduction of new colleagues. The decision by the departments to continuously invest resources into SDM training and education, updates of decision aids etc. is both an important factor and a sign of appreciation of SDM. The intrinsic motivation to maintain SDM is further illustrated by the activities within the departments that exceeded the basic criteria required to renew the S2C certificate, such as ongoing scientific activity and public communication in cooperation with the National Competency Center for Shared Decision Making as well as the development of additional support models for patients regarding decision making.

Apart from the efforts within the hospital, an additional factor was established on the system level. In collaboration with the largest health insurance company in Germany, the Techniker Krankenkasse (TK), we managed to put in place a reimbursement scheme triggering an additional fee for every patient case within a department that is awarded with the S2C certificate at University Hospital Schleswig-Holstein. The motivation of the TK was to increase patient safety through enhancement of SDM, as it is recommended by the WHO (31). The reimbursement partly covers the costs for the professional SDM trainers, the updates of the decision aids etc. It is important to know that currently the departments have no financial benefits from this reimbursement; on the contrary, they invest own resources in terms of working hours of their staff. This corroborates their intrinsic motivation to maintain SDM by continuation of the S2C program. The amount of time needed to maintain SDM on a level that is sufficient for regular recertification is, however, much smaller than during the initial implementation phase.

Some possible limitations of this study have to be discussed. Firstly, the question may arise whether the results sustainability within two departments are sufficiently representative for the entire hospital. On the one hand, the Neuromedical Center is a large-volume center within the hospital, with a broad variety of diseases, treatments and patient characteristics. On the other hand, the S2C program has been successfully implemented in 15 other departments at the University Hospital Schleswig-Holstein simultaneously also using the standardized S2C approach (publications in preparation). There were no cues indicating that the implementation process in the Neuromedical Center was considerably different compared to the other departments. The reason the Neuromedical Center was chosen for examination of sustainability is that it was the first entire center that had started and fully completed the intervention. This allowed for the longest follow-up period during the overarching implementation project (24). Hence, it seems justified to interpret the results as proof of the sustainability of the S2C program in general. However, future results from other departments, and from other hospitals, should be gathered to underscore this conclusion.

Secondly, data might be biased by self-selection of responding patients. However, response rates of around 60% are to be viewed as comparably high indicating a rather low risk of selection bias. In addition, neither physicians nor the study group had any influence on the selection of patients enrolled in this study.

Thirdly, all outcome data on sustainability reflect the patients' point of view using PICS, PrepDM, and CollaboRATE as retrospective patient reported outcome instruments. It is indisputable, that the patient's experience is of major importance, especially when other patient-related variables like e.g., adherence are discussed. Nevertheless, the evaluation within other departments at the UKSH in Kiel also includes observer-based analyses of videotaped consultations using MAPPIN'SDM (32) and data on costs and quality of care as a result of the SDM implementation (24). With those future findings available, it will be possible to further corroborate the conclusions drawn from the current study.

Fourthly, data might also be biased by the occurrence of the Covid-19 pandemic. While the pre-intervention data (*t*_0_) were collected during regular hospital operation, the long-term (*t*_2_) data collection took place from May 2021 on, immediately after a multi-week lockdown for the majority of the German population. During this period, elective procedures and treatments were restricted or postponed, so that the patient sample must be expected to be different from the pre-intervention sample. However, a reduction of elective treatments at *t*_2_ should result in a *lower* level of perceived SDM, not a higher one. Therefore, the positive effect in this study can hardly be explained by the influence of the pandemic. On the contrary, the pandemic in general made it even harder for all departments to adhere to the intervention program. Nevertheless, in view of the major influence of the pandemic on all levels of healthcare, post-pandemic replications of these findings are welcome.

Fifthly, in subsequent analyses of this study, we found a potential impact of age on both indicators of the SDM level indicating a smaller long-term effect among older patients. However, the size of some of the compared subgroups is very small. As such effects had not been found in previous data from the same population (21), the slightly differential effect among the subgroups should be interpreted cautiously until more long-term data are available.

In conclusion, this study is valuable as it provides long-term results from a hospital wide SDM implementation effort. It shows that the comprehensive, multifaceted S2C program has significant long-term effects on patient reported SDM and health literacy by inducing sustained intervention effects and the willingness among health professionals to actively maintain the SDM implementation. Future results from the ongoing S2C program in Kiel and in other hospitals will further broaden the knowledge on the sustainability of the program.

## Data availability statement

The raw data supporting the conclusions of this article will be made available by the authors, without undue reservation.

## Ethics statement

The study was approved by the Ethics Committee of Faculty of Medicine of Kiel University (reference number A111/18).

## Author contributions

FG, KW, FS, JR, LS, and MLC contributed to conception and design of the study. CS-K and CB organized the database. LS, MC, and WS performed the statistical analysis. CS-K wrote the first draft of the manuscript. FG, FS, CB, and CS-K wrote sections of the manuscript. All authors contributed to manuscript revision, read, and approved the submitted version.

## Funding

The SHARE TO CARE project was funded by the German Innovation Fund of the Federal Joint Committee (NVF170009) and the Medical Faculty of the Kiel University.

## Conflict of interest

FG and FS are co-founders, JR is CEO and co-funder of SHARE TO CARE Patientenzentrierte Versorgung GmbH (Cologne/Germany). JR is CEO of TakePart Media + Science GmbH, Cologne, Germany. The remaining authors declare that the research was conducted in the absence of any commercial or financial relationships that could be construed as a potential conflict of interest.

## Publisher's note

All claims expressed in this article are solely those of the authors and do not necessarily represent those of their affiliated organizations, or those of the publisher, the editors and the reviewers. Any product that may be evaluated in this article, or claim that may be made by its manufacturer, is not guaranteed or endorsed by the publisher.
